# Prediction of the Active Components and Mechanism of *Forsythia suspensa* Leaf against Respiratory Syncytial Virus Based on Network Pharmacology

**DOI:** 10.1155/2022/5643345

**Published:** 2022-07-20

**Authors:** Xiaoxue Wang, Ping Wang, Haitao Du, Na Li, Tianyuan Jing, Ru Zhang, Wanying Qi, Yanan Hu, Tianyu Liu, Lanxin Zhang, Nan Xu, Yi Wang, Huimin Zhang, Xiaoyan Ding

**Affiliations:** ^1^College of Pharmacy, Shandong University of Traditional Chinese Medicine, Jinan, Shandong, China; ^2^Shandong Academy of Chinese Medicine, Jinan, Shandong, China; ^3^State Key Laboratory of Precision Testing Technology and Instruments, Tianjin University, Tianjin, China; ^4^Shanghai International Studies University, Shanghai, China; ^5^Macau University of Science and Technology, Macau, China

## Abstract

**Objective:**

*Forsythia suspensa* leaf (FSL) has been used as a health tea in China for centuries. Previous experiments have proved that FSL extract has a good effect on the antirespiratory syncytial virus (RSV) *in vitro*, but its exact mechanism is not clear. Therefore, this study aims to determine the active components and targets of FSL and further explore its anti-RSV mechanism.

**Methods:**

UPLC-Q-Exactive-MS was used to analyze the main chemical components of FSL. The compound disease target network, PPI, GO, and KEGG were used to obtain key targets and potential ways. Then, the molecular docking was verified by Schrödinger Maestro software. Next, the cell model of RSV infection was established, and the inhibitory effect of each drug on RSV was detected. Finally, western blotting was used to detect the effect of the active components of FSL on the expression of PI3K/AKT signaling pathway-related protein.

**Results:**

UPLC-Q-Exactive-MS analysis showed that there were 67 main chemical constituents in FSL, while network pharmacological analysis showed that there were 169 anti-RSV targets of the active components in FSL, involving 177 signal pathways, among which PI3K/AKT signal pathway played an important role in the anti-RSV process of FSL. The results of molecular docking showed that cryptochlorogenic acid, phillyrin, phillygenin, rutin, and rosmarinic acid had higher binding activities to TP53, STAT3, MAPK1, AKT1, and MAPK3, respectively. *In vitro* experiments showed that phillyrin and rosmarinic acid could effectively improve the survival rate of RSV-infected cells, increase the expression level of PI3K, and decrease the expression level of AKT.

**Conclusion:**

The active ingredients of FSL, phillyrin, and rosmarinic acid can play an anti-RSV role by inhibiting PI3K/AKT signaling pathway. This study provides reliable theoretical and experimental support for the anti-RSV treatment of FSL.

## 1. Introduction

The vulnerable part of the human body is the respiratory tract, most of which are caused by viruses, so viral respiratory diseases have become the most common infectious diseases in human beings. The outbreak of COVID-19 at the end of 2019 clearly shows the high risk of viral infection. Respiratory syncytial virus (RSV), one of the most common respiratory viruses, is named as it can cause fusion lesions in respiratory tissue culture cells [1]. RSV is a negative single-stranded RNA virus of the family Paramyxoviridae, and is also the main pathogen of early acute respiratory infection in children [2]. Epidemiological investigation shows that RSV infection has certain seasonality. Infants, children, and elderly people with low immunity are vulnerable to infection. Without specific immunity after infection, there is a risk of reinfection [2–4]. More than 30 million children in the world are infected with RSV each year, but there is no specific therapeutic drug or preventive vaccine at present. Ribavirin, a broad-spectrum antiviral drug, has a certain inhibitory effect on RSV, however, with serious adverse reactions, such as myelosuppression and carcinogenicity; it is limited in clinical use [5].

Traditional Chinese medicine has long played a unique role in the treatment of viral infectious diseases because of its multicomponent and multitarget advantages. Traditional heat-clearing drugs, including Scutellaria baicalensis, Coptis chinensis and Forsythia suspensa have been proved to have a significant anti-RSV effect [6–8]. In recent years, studies have shown that the chemical constituents in the leaves and fruits of Forsythia suspensa are very similar, and the content of phillyrin in the leaves of Forsythia is even 40 times higher than that in the fruits of *Forsythia suspensa* [9], which reveals that the leaves of Forsythia suspensa have a potential anti-RSV effect. *Forsythia suspensa* leaf (FSL) comes from the compendium of materia medica, has a bitter taste, cold nature, and the effect of heat-clearing and detoxification. In addition, in the Yuqiao Medical Order, it is written that FSL is bitter, slightly pungent, cold in nature, can be used to relieve fire, clear heat, and benefit the heart and lung meridians. At present, more than 200 chemical constituents have been isolated and extracted from FSL. The main active components are phillyrin, phillygenin, rutin, chlorogenic acid, and caffeic acid, they have pharmacological effects, for instance, antibacterial, antiviral, liver protection, heart protection, antioxidation and so on [10–13]. *Forsythia suspensa* is usually used in medicine with fruit, which has a wide range of functions and a long history in medicine. Nevertheless, a large number of FSLs are discarded. The development and utilization of FSL are limited to the application of Forsythia tea in a small number of areas [14], which is a great waste of resources. Therefore, exploring the anti-RSV mechanism of the active components in FSL is not only conducive to the research and development of new anti-RSV drugs, but also provides a theoretical basis for the development and utilization of FSL.

Network pharmacology is a new research tool that comprehensively analyzes drugs, disease-related genes, and proteins in a bioinformatics database. It has been widely used in the research of traditional Chinese medicine, and it is of great significance to reveal the action mechanism of active ingredients of traditional Chinese medicine [15]. In addition, molecular docking is a means of further analysis based on the prediction of network pharmacology, which can study the interaction between ligand and receptor by simulating the interaction between molecules and the binding mode of both, so as to evaluate the binding potential of protein ligand [16]. Nowadays, using network pharmacology to clarify the potential mechanism of complex components through big data analysis and molecular docking simulation has become a common method to study the pharmacological mechanism of traditional Chinese medicine. Previous experiments conducted by our research group showed that the extract of FSL had a positive anti-RSV effect *in vitro*. In this study, the possible molecular mechanism between chemical components and disease targets of FSL was explored by network pharmacology, verified by molecular docking and *in vitro* experiments, and its action mechanism was explored by Western blot, providing a scientific basis for the further study of the material basis and molecular mechanism of anti-RSV in FSL.

## 2. Material and Methods

### 2.1. Chemical Composition Analysis of FSL

FSL was harvested from Shandong University of Traditional Chinese Medicine in April and identified by researcher Lin Huibin of Institute of Traditional Chinese Medicine Resources, Shandong Academy of Chinese Medicine as the leaves of *Forsythia suspensa* (Thunb.) Vahl, family Lignanaceae. The leaves were dried at room temperature, crushed, and preserved. Weighing 20 g FSL and adding water (1 : 10) to soak them for 2 h, then heating and refluxing twice, 1 h for each. The extract was combined, filtered, and concentrated to 1 g/mL as the original medicinal material, and stored in the refrigerator at −20°C. 1 mL solution was added to methanol at a constant volume of 100 mL. The supernatant was obtained by ultrasonic for 30 min, 6000 rpm centrifugation for 10 min, and then passed through a 0.22 *μ*m filter membrane. The chemical constituents of FSL were analyzed by UPLC-Q-Exactive-MS. In comparison to the literature, the compounds obtained were qualitatively analyzed, and the main chemical constituents of FSL were screened.

### 2.2. Target Acquisition of FSL

Firstly, the information of compounds and molecules was obtained by the PubChem database [17, 18] (https://pubchem.ncbi.nlm.nih.gov/), and the smile information of compounds was uploaded to the Swiss TargetPrediction database [19–21] (http://www.swisstargetprediction.ch/) to predict the target of compounds. UniProt database [22, 23] (https://www.uniprot.org/) was used to normalize the gene information, and the target with Probability > 0 was selected for the study. Comparing the target genes of the main active components of FSL with the targets related to Respiratory Syncytial Virus found in databases such as GeneCards [24, 25] (http://www.genecards.org/) and OMIM [26, 27] (https://omim.org/), the obtained repeated targets are the potential targets of the main active components of FSL against the respiratory syncytial virus. Using Cytoscape software (version 3.7.1) [28, 29], the network diagram of active components-targets of FSL was constructed.

### 2.3. Construction of Compound Targeting Pathway Network of FSL

The potential targets of FSL against RSV were introduced into the string platform [30, 31] (https://string-db.org/) to construct a functional PPI analysis (confidence is 0.7). Then, KEGG pathway analysis and gene ontology biological process analysis were carried out by DAVID Bioinformatics Resources (https://david.ncifcrf.gov/) [32, 33], and the correlation analysis results were obtained (*P* < 0.05). The KEGG pathway analysis results were visualized by using OmicShare tools [34,35].

### 2.4. Molecular Docking

Firstly, 3D structures of 67 chemical components were obtained from the PubChem database. Then, using the PDB database (https://www.rcsb.org/) and Uniprot database (https://www.uniprot.org/) [36], the target protein crystal structure TP53 with high resolution (Å) and complete structure TP53 (PDB ID : 1YC5, The resolution is 1.4 Å), STAT3(PDB ID : 6NJS, resolution 2.70 Å), MAPK1(PDB ID : 2Y9Q, resolution 1.55 Å), AKT1(PDB ID : 1UNQ, resolution 0.98 Å) and MAPK3(PDB ID : 4QTB, resolution 1.40 Å). Molecular docking was performed by Schrödinger Maestro software (version free) [37]. Use LigPrep and Protein Preparation Wizard plug-ins to pretreat ligand molecules and target proteins, hydrogenate proteins, remove water molecules, and charge them; The receptor grid generation plug-in is used to generate the target protein grid file, and all amino acid residues within the radius of 10 are used as active cavities; Finally, the ligand docking plug-in is used to dock the ligand molecule with the target protein, and the docking diagram and docking score are output to evaluate the docking of the receptor and the ligand.

### 2.5. Cells and Viruses

Respiratory syncytial viruses (RSV, Institute of Basic Medical College, Shandong Academy of Medical Sciences, Shandong, China) were amplified from human laryngeal epidermoid cancer cells (HEp-2; Institute of Basic Medical College, Shandong Academy of Medical Sciences, Shandong, China). The cells were cultured in a humidified incubator (5% CO_2_, 37°C) with a DMEM medium containing 10% fetal bovine serum and antibiotics. 50% histiocytic infection dose (TCID_50_) of the virus was determined. The virus was stored in a refrigerator at −80°C.

### 2.6. Anti-RSV Efficacy of the Active Ingredients of Forsythia Suspense Leaf

Taking a 96-well plate covered with monolayer HEp-2 cells, five standard drugs (five compounds with the best molecular docking fraction in 2.5) with 2 times dilution, water extract of FSL and ribavirin were added to the plate. Normal cells were set as control and cultured at 37°C in a 5%CO_2_ incubator for 24 h. MTT [38] (Thiazolyl Blue Tetrazolium Bromide, 3-(4,5-Dimethylthiazol-2-yl)-2,5-diphenyltetrazolium bromide) was used to measure OD_490 nm_ and determine the maximum nontoxic concentration of each drug.

The 96-well plates full of monolayer HEp-2 cells were divided into the normal group, model group, ribavirin group, and drug group. Except for the normal group, the other groups were added with 50 *μ*L of RSV virus of 100TCID_50_. In the drug group, 50 *μ*L of each series of drugs diluted by 2 times were added, with a total of 8 concentrations, and 6 multiple wells were set up. In the virus group and the normal group, 2% cell maintenance solution, 50 *μ*L/well, and 100 *μ*L/well were added, respectively. They were cultured in an incubator and observed daily, and the OD_490 nm_ value was measured by MTT at 24 and 48 hours after infection. The cell survival rate of each drug group was calculated.

### 2.7. Effect of Drugs on PI3K/AKT Signaling Pathway in HEp-2 Cells by Western Blot

The HEp-2 cells of each group were collected, and the total protein was extracted with RIPA lysate (Solarbio, Beijing, China). The standard curve was drawn, and the protein content was determined according to the instructions of the BCA kit (Solarbio, Beijing, China). Based on the differences in the relative molecular weight of proteins, 7.5%–12.5% SDS-PAGE gel electrophoresis was performed. After electrophoresis, the protein was transferred to the PVDF membrane, sealed in 5% defatted milk powder at room temperature for 3 h, and the target molecule-specific primary antibodies were incubated overnight at 4°C. The first antibodies included PI3K (1 : 1000), *p*-PI3K (1 : 1000), AKT (1 : 2000), *p*-AKT (1 : 1500) (Abcam, UK), *β*-Actin (1 : 1000) (Cell Signaling Technology, USA). After 1 × TBST washing, antirabbit IgG labeled with horseradish peroxidase (Zhongshanjinqiao, Beijing, China) was incubated at room temperature for 1 h. The protein bands were developed with ECL photoluminescence solution, and the experimental results were analyzed by Image J software (version 1.8.0) [39].

### 2.8. Statistical Analysis

The experimental data were analyzed by IBM SPSS Statistics software (version 28.0) [40]. Each independent experiment was repeated at least three times to obtain the average value, and the data were expressed as the mean ± standard deviation. One-way ANOVA and LSD multiple comparison analysis were used for comparison among multiple groups, and *P* < 0.05 indicated that the difference was statistically significant.

## 3. Result

### 3.1. Determination of Chemical Constituents of FSL

UPLC-Q-Exactive-MS was used to identify and analyze the main chemical components of FSL water extract. A total of 67 compounds were obtained by comparing the relative molecular weight information of compounds provided by mass spectrometry with known databases and references, it mainly includes caffeic acid, phillyrin, (+) - rosin-beta-d-glucopyranoside, calceolarioside B, forsythin glycoside B, and rutin (Table 1).

### 3.2. Active Ingredient Targets Prediction

**Figure 1:**
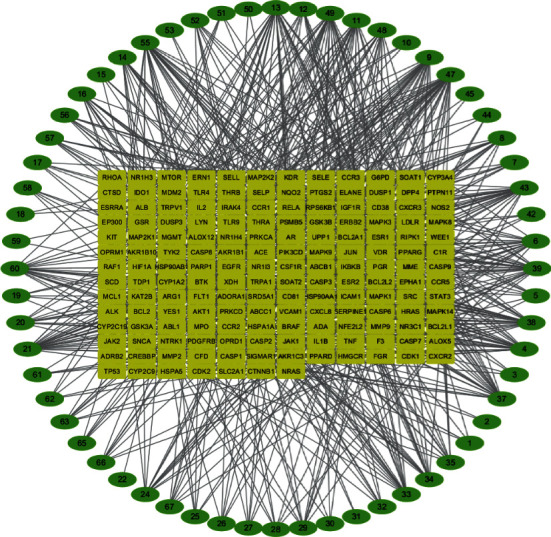
Active ingredient of FSL-RSV pneumonia target network.

In this study, a total of 2952 potential targets for FSL and 849 RSV disease targets were obtained. After comparison and analysis with potential targets for FSL, 169 potential targets for anti-RSV of FSL were obtained, mainly including matrix metalloproteinase 2 (MMP2), mitogen-activated protein kinase activated protein kinase 2 (MAPKAPK2), and cyclin-dependent kinase 1 (CDK1). The intersection targets and their component relationships are imported into Cytoscape 3.7.1 for visualization, forming a network of “active components of FSL-RSV pneumonia targets.” The gene network has 223 nodes and 683 edges in total (Figure 1).

### 3.3. Structure Protein-Protein Interaction (PPI) Network

**Figure 2:**
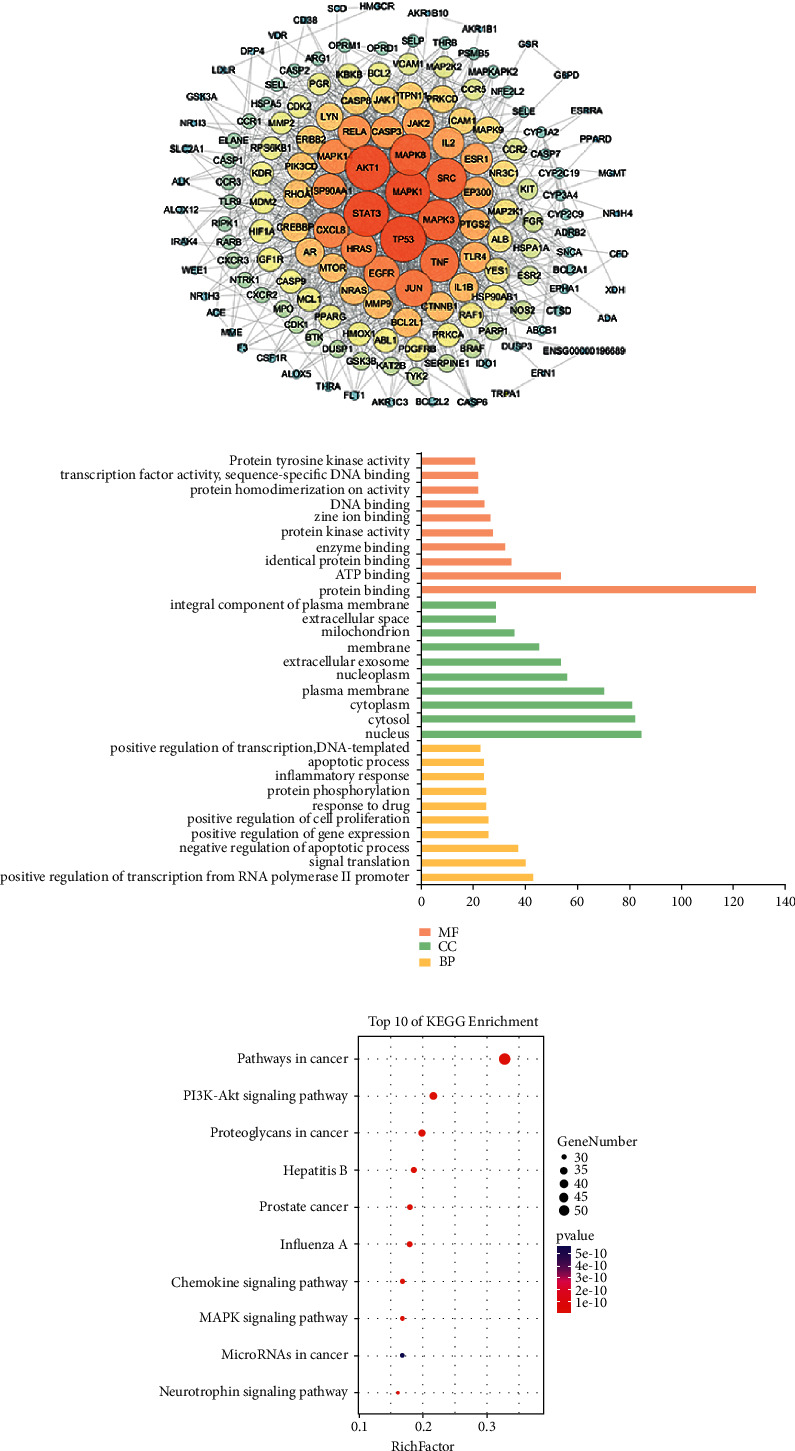
(a) PPI network of FSL anti-RSV; (b) GO enrichment analysis of anti-RSV target biological processes of FSL; (c) bubble diagram of KEGG enrichment pathway, the main target of FSL.

Nodes were evaluated and colored by node degree values, the anti-RSV protein-protein interaction network diagram of FSL was obtained (Figure 2(a)). The network diagram contains 155 targets and 1330 edges. The larger the degree value is, the larger the node is. The protein with a large degree value (degree value > 50) is TP53, STAT3, MAPK1, AKT1, MAPK3, MAPK8, SRC, and TNF. Through the PPI network diagram, we can intuitively discover the complex interactive relationship between compounds and targets, and further, characterize the synergistic regulation of anti-RSV by FSL by multicomponent and multitarget features.

The DAVID database was used to analyze the anti-RSV targets of the main components of FSL by GO and KEGG. The results of GO analysis show that FSL mainly takes effects by regulating the following biological processes: under the condition of *P* < 0.05, 475 BP (Bioprogress, cell biological processes, such as adhesion, migration, and apoptosis, cycle.), 59 CC (Cell Components, cell composition, such as cell membrane and cytoplasm.), 132 MF (Molecular Function) (Figure 2(b), ranked according to Count, the top 10), and 30 pathways were selected. There are 10 signaling pathways in KEGG analysis, among which Pathways in cancer, PI3K/AKT signaling pathway, Proteoglycans in cancer, Hepatitis B, and other four pathways have large nodes, which may be an important pathway for Forsythia suspense leaf to play an anti-RSV role. 10 pathways are visualized by sorting according to the number of target hits (Figure 2(c)).

### 3.4. Molecular Docking

**Figure 3:**
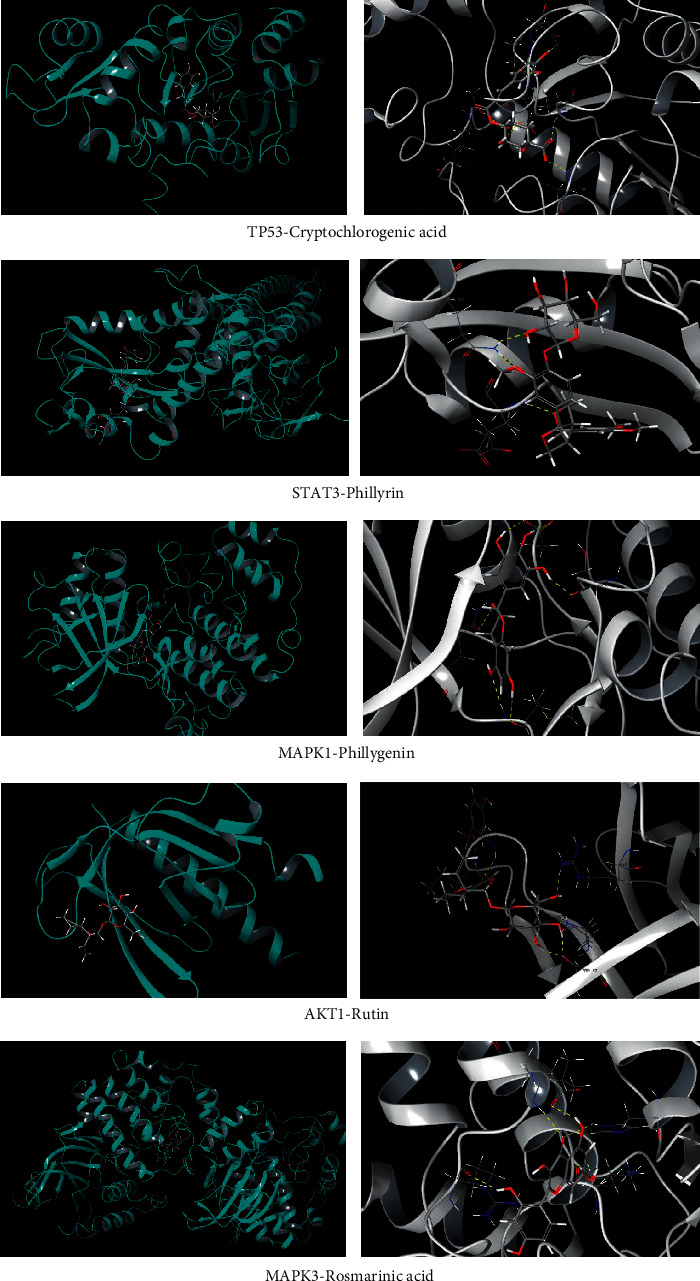
Molecular docking of active components of FSL with core targets. The yellow dotted line represents H-bond, and the green dotted line represents Pi-cation.

Schrödinger molecular docking module provides SP (standard precision) and XP (extra precision) to ensure the speed and accuracy of docking results. Moreover, the contribution of hydrogen bond, lipophilicity, and metal ligand, as well as the rotation of unsuitable bonds and atoms with steric repulsion will be fully considered in the docking score to ensure the reliability of the results. The 67 compounds selected under 2.1 were molecularly docked with five potential target proteins with network node (degree) > 56. The larger the absolute value of the docking score, the greater the affinity of docking between ligand and receptor. When the absolute value of docking score is greater than 5.0, it indicates that there is a strong interaction between the compound and the target protein, and the binding configuration of the compound and the target has a strong activity. The docking score results are shown in Table 2, which lists five compounds with high binding activity with five target proteins. The results showed that cryptochlorogenic acid, phillyrin, phillygenin, rutin, and rosmarinic acid had a high binding activity with TP53, STAT3, MAPK1, AKT1, and MAPK3, respectively. The docking mode diagram is shown in Figure 3. Therefore, cryptochlorogenic acid, phillyrin, phillygenin, rutin, and rosmarinic acid were selected for pharmacodynamic verification.

TP53, tumor protein p53; STAT3, signal transducer and activator of transcription 3; MAPK1, mitogen-activated protein kinase 1; AKT1, AKT serine/threonine kinase 1; MAPK3, mitogen-activated protein kinase 3.

### 3.5. Determination of the TCID_50_ of RSV and the Toxicity of Drugs to HEp-2

The determination of TCID_50_ is as follows: Calculate the TCID_50_ of RSV to be 10^−5.67^/100 *μ*L.

Determination of the maximum nontoxic concentration of the active ingredients of Forsythia suspensa leaf is as follows: The toxicity of the five compounds and the positive drug ribavirin to HEp-2 cells showed that most cells were broken or detached, and the cell growth rate was significantly reduced. Cell survival rate >90% was selected as the TC_0_ of each administration group on HEp-2 cells, and the TC_0_ of each administration group on HEp-2 cells was also measured (Table 3).

### 3.6. Anti-RSV Efficacy of the Active Ingredients of Forsythia Suspense Leaf

**Figure 4:**
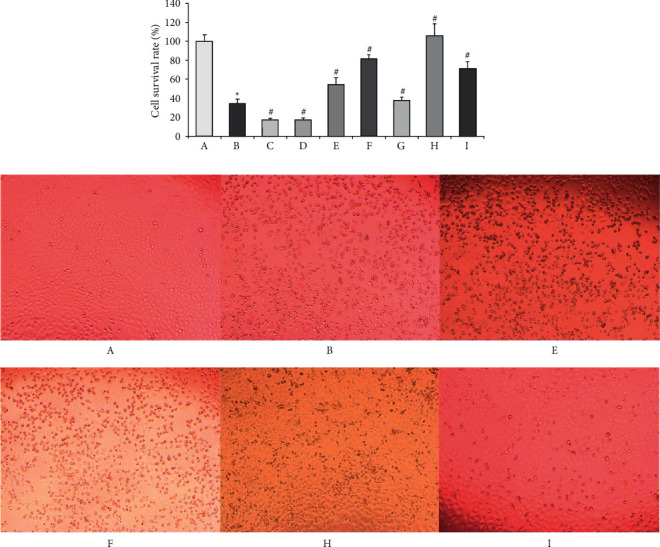
The effect of the active ingredients of FSL on the survival rate of RSV-infected HEp-2 cells(‾*x* ± *s*, *n* = 6) (a). Normal group; (b) model group; (c) 125 *μ*g/mL cryptochlorogenic acid; (d) 62.5 *μ*g/mL *phillyringenin*; (e) 1000 *μ*g/mL *phillyrin*; (f) 125 *μ*g/mL rosmarinic acid; (g) 62.5 *μ*g/mL rutin; (h) 2500 *μ*g/mL water extract of FSL; (I) 250 *μ*g/mL ribavirin; compared with normal group^*∗*^*P* < 0.05; compared with model group ^#^*P* < 0.05.

Compared with the normal group, the OD value of the model group was significantly reduced at 24 h and 48 h after RSV infected, and the HEp-2 cells had obvious lesions; compared with the model group, the OD value and the number of cells increased significantly at each concentration of each drug group and ribavirin group at 24 h, but after 48 h, there was no significant difference between each administration group and model group. The 24 h cell survival rate showed that the rate of the model group was (21.62 ± 4.40) %, significantly lower than that of the normal group (^*∗*^*P* < 0.05). Compared with the model group, the cell survival rates in the phillyrin and rosmarinic acid groups were significantly increased (^#^*P* < 0.05), indicating that the effective components of phillyrin and rosmarinic acid in Forsythia suspense leaf could improve the cell survival rate of RSV infection and effectively inhibit the proliferation of RSV(Figure 4). According to the results of the enrichment pathway in 3.3, phillyrin and rosmarinic acid were selected for Western blot verification of PI3K/AKT channel protein.

### 3.7. Effect of Drugs on PI3K/AKT Signaling Pathway in HEp-2 Cells by Western Blot

**Figure 5:**
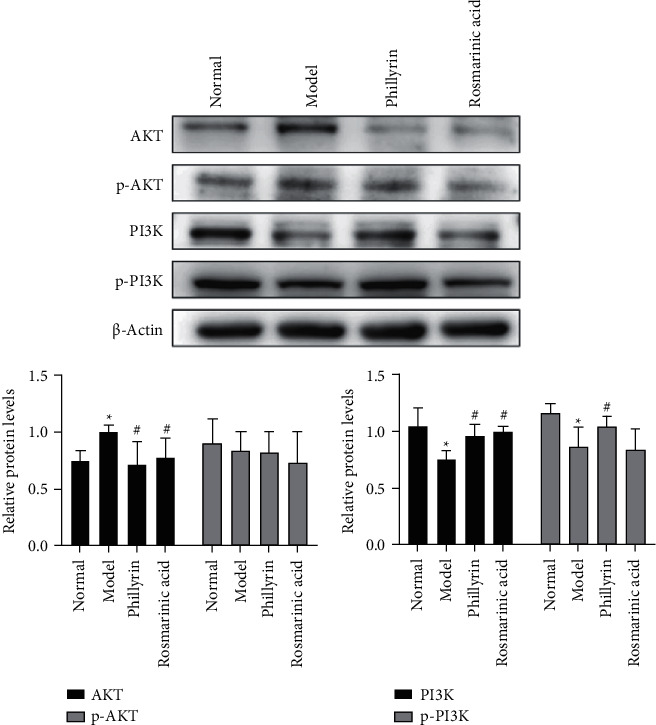
Effects of rosmarinic acid and *phillyrin* on PI3K/AKT signaling pathway-related proteins ( ‾*x* ± *s*, *n* = 3).

Compared with the normal group, the relative expression level of AKT in the model group was significantly increased(^*∗*^*P* < 0.05), and the relative expression levels of PI3K and p-PI3K were significantly decreased (^*∗*^*P* < 0.05). Compared with the model group, the relative expression level of AKT in the phillyrin group was significantly decreased (^#^*P* < 0.05), and the relative expression levels of PI3K and p-PI3K were significantly increased (^#^*P* < 0.05). The relative expression level of AKT in the Rosmarinic acid group was significantly decreased (^#^*P* < 0.05), and the relative expression level of PI3K was significantly increased (^#^*P* < 0.05) (Figure 5).

## 4. Discussion

RSV is the most common pathogen of viral pneumonia in children, which can cause interstitial pneumonia, bronchiolitis, and other inflammatory diseases, and reinfection is very common, unfortunately, there is still no approved RSV vaccine in China. In recent years, studies on traditional Chinese medicine and its preparations have shown that traditional Chinese medicine has unique advantages in antiviral owing to its many action targets and low incidence of drug resistance. [41] In China, FSL has a long history as tea. It has various chemical components, including phenyl ethanol and its glycosides, lignans and its glycosides, volatile oil, and other chemical substances, [42] among them, a variety of traditional Chinese medicine preparations containing phillyrin have been proved to have significant anti-RSV effects, which reveal that FSL has potential anti-RSV value. In this study, the combination of network pharmacology and experimental verification was used to explore the active components and mechanism of anti-RSV.

In this paper, the method of network pharmacology combined with molecular docking was used to explore the complex network of multicomponent, multitarget, and multipathway of anti-RSV processes in FSL. Firstly, 67 compounds were isolated and identified from the decoction of FSL by LC-MS, and then 2952 targets of FSL, 849 targets of RSV pneumonia, and 169 targets of the anti-RSV potential of the active components in FSL were screened. The results of the PPI network analysis showed that FSL may play an anti-RSV effect by regulating target proteins, for example, TP53, STAT3, MAPK1, AKT1, MAPK3, MAPK8, SRC, TNF, and so on. GO enrichment analysis showed that the biological process of RSV resistance of FSL involved protein binding, nucleus, cytosol, cytoplasm, and plasma membrane. KEGG pathway enrichment analysis showed that pathways in cancer, PI3K/AKT signaling pathway, proteoglycans in cancer, and hepatitis B pathways played an important role. The results of docking between the active components of FSL and the main target protein molecules showed that cryptochlorogenic acid, phillyrin, phillyringenin, rutin, and rosmarinic acid had higher binding activities to TP53, STAT3, MAPK1, AKT1, and MAPK3, respectively. *In vitro* antiviral experiments of water extract of FSL and five active components in FSL show that water extract of FSL and active components phillyrin and rosmarinic acid have obvious RSV inhibition effect, and the anti-RSV effect of water extract of FSL is better than that of the two active components. It is speculated that the antiviral effect of FSL is the result of the interaction and synergy of various active components.

**Figure 6:**
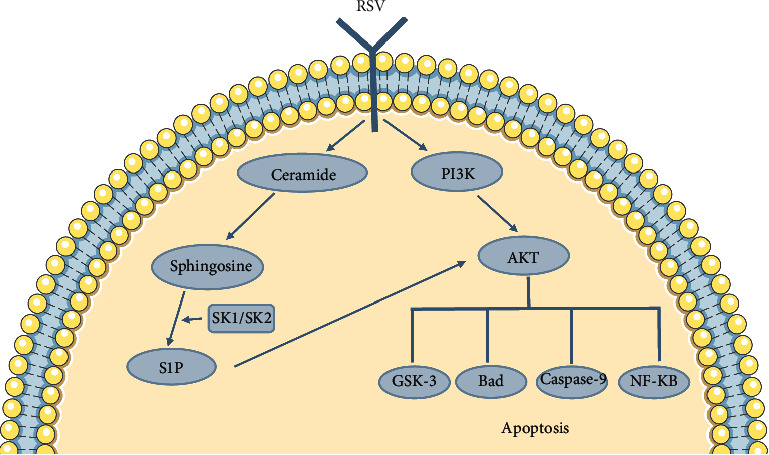
Model for prolonged cell survival after RSV infection.

According to the results of GO enrichment analysis and KEGG pathway enrichment analysis, the PI3K/AKT signal pathway may have potential value in the anti-RSV treatment of FSL; therefore, PI3K/AKT signal pathway is selected for further study. Western blotting experiments show that the relative expression level of AKT in the phillyrin group was significantly decreased, while the relative expression level of PI3K and p-PI3K was significantly increased. The relative expression level of AKT in the rosmarinic acid group decreased significantly, while the relative expression level of PI3K increased significantly, suggesting that the anti-RSV effect of FSL may be related to the inhibition of the PI3K/AKT pathway. Studies have shown that the PI3K/AKT signal pathway plays an important regulatory role in pulmonary inflammatory diseases. Resveratrol can inhibit the activation of the PI3K/AKT signal pathway induced by RSV, reduce the inflammatory response, and thus play a protective role in lung injury caused by RSV. PI3K/AKT pathway is a vital signal transduction pathway in cells, which can regulate cell differentiation, proliferation, activation, and antiviral ability through the expression of a series of protein factors [43, 44]. PI3K (phosphatidylinositol-3-kinase) is a dimer composed of regulatory subunit p85 and catalytic subunit p110. After activation, a second messenger PIP3 (phosphatidylinositol-3-phosphate) is produced on the plasma membrane. PIP3 can change the protein structure of AKT and activate it, and activate or inhibit the activity of a series of downstream substrates, such as apoptosis-related proteins Bad and caspase 9 by phosphorylation, thereby regulating cell proliferation, differentiation, apoptosis, and migration. [45, 46] Therefore, the activation of the PI3K/AKT signal pathway can reduce the effect of apoptosis and prolong the survival time of cells. On the one hand, RSV infection can up-regulate S1P (sphingosine 1-phosphate) [47], which can mediate the activation of the AKT pathway and up-regulate the expression of the Mdm-2 gene, thus mediating the degradation of tumor suppressor gene p53 and promoting the cell survival time. On the other hand, it can also directly activate PI3K/AKT signaling pathway and inhibit cell apoptosis [48] (Figure 6), thus facilitating the virus to complete its own life cycle for amplification before cell apoptosis. [49, 50] It can be speculated that the active components in FSL promote apoptosis and shorten cell survival time by inhibiting PI3K/AKT signal pathway, so as to play an anti-RSV role.

The present study has several limitations. Firstly, due to the continuous updating of the network pharmacology database, there may be a lack of up-to-date bioactive components and target genes in this study. In addition, other signal pathways may also be involved in the anti-RSV effect of active components in FSL, which needs to be verified by more experiments. Finally, the solubility of phillyrin reduces its efficacy *in vitro*, and more studies are needed to further explore the molecular mechanism of phillyrin against RSV in *vivo*.

## 5. Conclusion

To sum up, based on network pharmacology combined with the molecular docking method, this study explored the active ingredients and potential mechanism of FSL against RSV through *in vitro* experiments. The results showed that phillyrin and rosmarinic acid in FSL could inhibit PI3K/AKT signaling pathway, promote cell apoptosis and shorten cell survival time, thus playing an anti-RSV role. This study can not only provide a theoretical basis and experimental support for the further development and application of FSL and its effective components in the future but also provide a promising way to reveal the scientific basis and treatment mechanism of diseases treated by traditional Chinese medicine.

## Figures and Tables

**Table 1:** Main hemical composition of FSL.

NO.	Compound	Molecular formula	Ion mode	Mzmed	RT/min	Peak area
1	(+)-Pinoresinol	C_20_H_22_O_6_	[M-H]^−^	357.13	6.56	791229617.70
2	(+)-Pinoresinol-4-O-beta-D-glucopyraside	C_26_H_32_O_11_1	[M-H]^−^	519.19	6.56	4166509751.00
3	Salidroside	C_14_H_20_O_7_	[M-H]^−^	299.11	2.98	416030123.60
4	Quercetin	C_15_H_10_O_7_	[M + H]^+^	303.05	6.60	883627.57
5	Hyperoside	C_21_H_20_O_12_2	[M-H]^−^	463.08	6.08	7594792.25
6	Caffeic acid	C_9_H_8_O_4_	[M-H]^−^	179.03	4.01	10694238742.00
7	Phillyrin	C_27_H_34_O_11_1	[M-H]^−^	579.20	7.73	7458440880.00
8	Chlorogenic acid	C_16_H_18_O_9_	[M-H]^−^	353.08	3.57	110358675.20
9	Kaempferol	C_15_H_10_O_6_	[M-H]^−^	285.03	8.89	355385.01
10	3-Hydroxybenzaldehyde	C_7_H_6_O_2_	[M-H]^−^	121.02	4.21	188945020.50
11	Ferulic acid	C_10_H_10_O_4_	[M-H]^−^	193.04	4.49	4914965.43
12	P-Hydroxyphenyl acrylic acid	C_9_H_8_O_3_	[M-H]^−^	163.03	4.65	10697330.91
13	Secoisolariciresinol	C_20_H_26_O_6_	[M-H]^−^	361.16	7.09	9828539.83
14	Calceolarioside B	C_23_H_26_O_11_1	[M + H]^+^	479.16	5.89	675251118.64
15	Vanillic acid	C_8_H_8_O_4_	[M-H]^−^	167.03	3.94	48787513.71
16	4-Dicaffeoylquinic acid	C_16_H_18_O_9_	[M-H]^−^	353.08	3.88	80843996.30
17	Quinic acid	C_7_H_12_O_6_	[M-H]^−^	191.05	4.57	57466022.97
18	3,4-Dihydroxybenzaldehyde	C_7_H_6_O_3_	[M-H]^−^	137.02	4.00	60332307.59
19	Phillyrin B	C_34_H_44_O_19_9	[M-H]^−^	755.23	6.00	820467980.90
20	4-Hydroxycinnamic acid	C_9_H_8_O_3_	[M-H]^−^	163.03	5.27	250776002.80
21	Rhamnocitrin	C_20_H_22_O_6_	[M-H]^−^	357.13	27.22	22984449.73
22	P-hydroxybenzoxal	C_7_H_6_O_2_	[M-H]^+^	123.04	0.87	24287717.24
23	Benzoic acid	C_7_H_6_O_2_	[M + H]^+^	123.04	3.64	40801996.94
24	Isophorone	C_9_H_14_O	[M-H]^+^	139.11	2.79	116897843.36
25	Nivolumab	C_8_H_8_O_3_	[M + H]^+^	153.06	5.21	62982567.92
26	Camphor	C_10_H_16_O	[M + H]^+^	153.13	5.84	106102205.21
27	Tryptamine	C_10_H_12_N_2_	[M + H]^+^	161.11	26.80	7964958.56
28	2-Coumarin	C_9_H_8_O_3_	[M + H]^+^	165.06	3.83	43963794.17
29	P-Hydroxycinnamic acid	C_9_H_8_O_3_	[M + H]^+^	165.06	4.58	9310132.79
30	Paeonol	C_9_H_10_O_3_	[M + H]^+^	167.07	3.22	30106741.86
31	Homogentisic acid	C_8_H_8_O_4_	[M + H]^+^	169.05	1.24	89280908.15
32	Geranic acid	C_10_H_16_O_2_	[M + H]^+^	169.12	1.02	56272751.98
33	6,7-Dihydroxy-4-methylcoumarin	C_10_H_8_O_4_	[M + H]^+^	193.05	7.40	11318256.46
34	Scopolactone	C_10_H_8_O_4_	[M + H]^+^	193.05	0.45	6146412.70
35	Yokogawa ligustilide A	C_12_H_16_O_2_	[M + H]^+^	193.12	8.71	9992106.14
36	Dihydrokaempferol (colombian aglycone)	C_14_H_14_O_4_	[M + H]^+^	285.05	1.74	1054100.46
37	Propethrin	C_19_H_26_O_3_	[M + H]^+^	303.20	7.44	37631362.46
38	Sanguinarine	C_15_H_10_O_7_	[M + H]^+^	303.05	8.10	44076.10
39	Boswellic acid	C_20_H_30_O_2_	[M + H]^+^	303.23	12.14	3471892.58
40	(-)-Epigallocatechin	C_15_H_14_O_7_	[M + H]^+^	307.08	6.12	282532593.99
41	Gallocatechins	C_15_H_14_O_7_	[M + H]^+^	307.08	4.69	7539336.66
42	Caffeinol	C_20_H_28_O_3_	[M + H]^+^	317.21	10.82	33616062.68
43	Isorhamnetin	C_16_H_12_O_7_	[M + H]^+^	317.07	6.46	1930112.37
44	Ginkgoneolic acid	C_20_H_32_O_3_	[M + H]^+^	321.24	11.45	8274443.51
45	Isoform aconitin	C_20_H_27_NO3	[M + H]^+^	330.21	6.89	17743758.07
46	Columbin	C_20_H_22_O_6_	[M + H]^+^	359.15	6.13	15304281.36
47	Genistein B	C_19_H_18_O_7_	[M + H]^+^	359.11	1.14	98701853.52
48	Rosmarinic acid	C_18_H_16_O_8_	[M + H]^+^	361.09	4.57	400615.12
49	Curcumin	C_21_H_20_O_6_	[M + H]^+^	369.13	9.96	5345129.23
50	Aucubin	C_15_H_22_O_9_	[M + H]^+^	369.12	2.21	3112265.84
51	Forsytheins	C_21_H_24_O_6_	[M + H]^+^	373.17	7.78	81425864.68
52	Paraphyllin	C_22_H_33_NO4	[M + H]^+^	376.25	4.94	21317926.78
53	Loganic acid	C_16_H_24_O_10_0	[M + H]^+^	377.15	4.69	61961252.11
54	Eleutheroside	C_16_H_22_O_9_	[M + H]^+^	381.12	2.97	39828726.65
55	Pseudolaric acid C	C_21_H_26_O_7_	[M + H]^+^	391.18	8.97	33835218.13
56	Afzelin	C_21_H_20_O_10_0	[M + H]^+^	433.11	6.37	1241906.35
57	Astragalin	C_21_H_20_O_11_1	[M + H]^+^	449.11	6.19	5731468.55
58	Isosakuranin	C_22_H_24_O_10_0	[M + H]^+^	449.15	3.63	58302445.74
59	Asperuloside acid	C_18_H_24_O_12_2	[M + H]^+^	455.12	4.25	1549292.02
60	Berberine	C_26_H_30_O_7_	[M + H]^+^	455.20	7.44	653736.80
61	Forsythoside E	C_20_H_30_O_12_2	[M + H]^+^	463.18	3.20	119659925.90
62	Methylnissolin-3-O-glucoside	C_23_H_26_O_10_0	[M + H]^+^	463.16	6.75	6053442.72
63	Isoquercitrin	C_21_H_20_O_12_2	[M + H]^+^	465.10	6.10	35289037.57
64	Daphylloside	C_19_H_26_O_12_2	[M + H]^+^	469.13	5.22	125152971.49
65	Calceolarioside A	C_23_H_26_O_11_1	[M + H]^+^	501.14	3.20	29042008.47
66	Rutin	C_27_H_30_O_16_6	[M + H]^+^	611.16	5.92	799317923.21
67	Isorhamnetin-3-o-neohesperidin	C_28_H_32_O_16_6	[M + H]^+^	625.18	6.45	3038686.75

**Table 2:** FSL compounds-potential target proteins docking score.

Target	Compound	Docking Score
TP53 (PDB ID : 1YC5)	Cryptochlorogenic acid	−8.146
	Phenyl sthanol glucoside B	−7.619
	Rosmarinic acid	−7.617
	Phenethyl acetate	−7.608
	Aucubin	−7.533

STAT3 (PDB ID : 6NJS)	Phillyrin	−5.269
	P-Hydroxyphenyl acrylic	−4.966
	4-Hydroxycinnamic acid	−4.966
	Paeonol	−4.966
	Vanillic acid	−4.907

MAPK1(PDB ID : 2Y9Q)	Phillygenin	−8.627
	Phenethyl acetate	−8.602
	Cryptochlorogenic acid	−8.362
	Quercetin	−8.353
	Loganine acid	−8.206

AKT1(PDB ID: 1UNQ)	Rutin	−6.057
	Vanillic acid	−5.956
	Dextroquinic acid	−5.854
	Dihydrobehenyl alcohol	−5.756
	Methyl paraben	−5.611

*MAPK3(PDB ID: 4QTB)*	*Rosmarinic acid*	−7.383
	Phillyrin	−7.129
	Cryptochlorogenic acid	−6.783
	Phenethyl acetate	−6.758
	Salidroside	−6.744

**Table 3:** TC_0_ on HEp-2 cells in each administration group (*n* = 6).

Name	TC_0_（*μ*g/mL）
Cryptochlorogenic acid	125
Phillygenin	250
Phillyrin	1000
Rosmarinic acid	250
Rutin	500
Ribavirin	250

TC_0_, maximal atoxic concentration.

## Data Availability

All data used to support the findings of this study are included within the article.

## Supplementary Materials

Supplementary Materials Supplementary Table 1. Main chemical composition of FSL. The optimal binding energy location of the target protein.
