# Supplementary material for: Prediction of the Active Components and Mechanism of Forsythia suspensa Leaf against Respiratory Syncytial Virus Based on Network Pharmacology
Source: Evid Based Complement Alternat Med. 2022 Jul 20;2022:5643345. doi: 10.1155/2022/5643345 (PMC9328944; doi:10.1155/2022/5643345)
Supplement: Supplementary Materials — Supplementary Table 1. Main chemical composition of FSL. The optimal binding energy location of the target protein. [file 5643345.f1.zip › 5643345.f1/Supplementary (1).docx]

Supplementary table 1

Table1 Main Chemical composition of FSL

| NO. | Compound | Molecular formula | Ion mode | Mzmed | RT/min | Peak area |
| --- | --- | --- | --- | --- | --- | --- |
| 1 | (+)-Pinoresinol | C_20_H_22_O_6_ | [M-H]^-^ | 357.13 | 6.56 | 791229617.70 |
| 2 | (+)-Pinoresinol-4-O-beta-D-glucopyraside | C_26_H_32_O_11_1 | [M-H]^-^ | 519.19 | 6.56 | 4166509751.00 |
| 3 | Salidroside | C_14_H_20_O_7_ | [M-H]^-^ | 299.11 | 2.98 | 416030123.60 |
| 4 | Quercetin | C_15_H_10_O_7_ | [M+H]^+^ | 303.05 | 6.60 | 883627.57 |
| 5 | Hyperoside | C_21_H_20_O_12_2 | [M-H]^-^ | 463.08 | 6.08 | 7594792.25 |
| 6 | Caffeic acid | C_9_H_8_O_4_ | [M-H]^-^ | 179.03 | 4.01 | 10694238742.00 |
| 7 | Phillyrin | C_27_H_34_O_11_1 | [M-H]^-^ | 579.20 | 7.73 | 7458440880.00 |
| 8 | Chlorogenic acid | C_16_H_18_O_9_ | [M-H]^-^ | 353.08 | 3.57 | 110358675.20 |
| 9 | Kaempferol | C_15_H_10_O_6_ | [M-H]^-^ | 285.03 | 8.89 | 355385.01 |
| 10 | 3-Hydroxybenzaldehyde | C_7_H_6_O_2_ | [M-H]^-^ | 121.02 | 4.21 | 188945020.50 |
| 11 | Ferulic acid | C_10_H_10_O_4_ | [M-H]^-^ | 193.04 | 4.49 | 4914965.43 |
| 12 | P-hydroxyphenyl acrylic acid | C_9_H_8_O_3_ | [M-H]^-^ | 163.03 | 4.65 | 10697330.91 |
| 13 | Secoisolariciresinol | C_20_H_26_O_6_ | [M-H]^-^ | 361.16 | 7.09 | 9828539.83 |
| 14 | Calceolarioside B | C_23_H_26_O_11_1 | [M+H]^+^ | 479.16 | 5.89 | 675251118.64 |
| 15 | Vanillic acid | C_8_H_8_O_4_ | [M-H]^-^ | 167.03 | 3.94 | 48787513.71 |
| 16 | 4-Dicaffeoylquinic Acid | C_16_H_18_O_9_ | [M-H]^-^ | 353.08 | 3.88 | 80843996.30 |
| 17 | Quinic acid | C_7_H_12_O_6_ | [M-H]^-^ | 191.05 | 4.57 | 57466022.97 |
| 18 | 3,4-Dihydroxybenzaldehyde | C_7_H_6_O_3_ | [M-H]^-^ | 137.02 | 4.00 | 60332307.59 |
| 19 | Phillyrin B | C_34_H_44_O_19_9 | [M-H]^-^ | 755.23 | 6.00 | 820467980.90 |
| 20 | 4-Hydroxycinnamic acid | C_9_H_8_O_3_ | [M-H]^-^ | 163.03 | 5.27 | 250776002.80 |
| 21 | Rhamnocitrin | C_20_H_22_O_6_ | [M-H]^-^ | 357.13 | 27.22 | 22984449.73 |
| 22 | P-hydroxybenzoxal | C_7_H_6_O_2_ | [M+H]^+^ | 123.04 | 0.87 | 24287717.24 |
| 23 | Benzoic acid | C_7_H_6_O_2_ | [M+H]^+^ | 123.04 | 3.64 | 40801996.94 |
| 24 | Isophorone | C_9_H_14_O | [M+H]^+^ | 139.11 | 2.79 | 116897843.36 |
| 25 | Nivolumab | C_8_H_8_O_3_ | [M+H]^+^ | 153.06 | 5.21 | 62982567.92 |
| 26 | Camphor | C_10_H_16_O | [M+H]^+^ | 153.13 | 5.84 | 106102205.21 |
| 27 | Tryptamine | C_10_H_12_N_2_ | [M+H]^+^ | 161.11 | 26.80 | 7964958.56 |
| 28 | 2-Coumarin | C_9_H_8_O_3_ | [M+H]^+^ | 165.06 | 3.83 | 43963794.17 |
| 29 | P-hydroxycinnamic acid | C_9_H_8_O_3_ | [M+H]^+^ | 165.06 | 4.58 | 9310132.79 |
| 30 | Paeonol | C_9_H_10_O_3_ | [M+H]^+^ | 167.07 | 3.22 | 30106741.86 |
| 31 | Homogentisic acid | C_8_H_8_O_4_ | [M+H]^+^ | 169.05 | 1.24 | 89280908.15 |
| 32 | Geranic acid | C_10_H_16_O_2_ | [M+H]^+^ | 169.12 | 1.02 | 56272751.98 |
| 33 | 6,7-Dihydroxy-4-methylcoumarin | C_10_H_8_O_4_ | [M+H]^+^ | 193.05 | 7.40 | 11318256.46 |
| 34 | Scopolactone | C_10_H_8_O_4_ | [M+H]^+^ | 193.05 | 0.45 | 6146412.70 |
| 35 | Yokogawa ligustilide A | C_12_H_16_O_2_ | [M+H]^+^ | 193.12 | 8.71 | 9992106.14 |
| 36 | Dihydrokaempferol (Colombian aglycone) | C_14_H_14_O_4_ | [M+H]^+^ | 285.05 | 1.74 | 1054100.46 |
| 37 | Propethrin | C_19_H_26_O_3_ | [M+H]^+^ | 303.20 | 7.44 | 37631362.46 |
| 38 | Sanguinarine | C_15_H_10_O_7_ | [M+H]^+^ | 303.05 | 8.10 | 44076.10 |
| 39 | Boswellic acid | C_20_H_30_O_2_ | [M+H]^+^ | 303.23 | 12.14 | 3471892.58 |
| 40 | (-)-Epigallocatechin | C_15_H_14_O_7_ | [M+H]^+^ | 307.08 | 6.12 | 282532593.99 |
| 41 | Gallocatechins | C_15_H_14_O_7_ | [M+H]^+^ | 307.08 | 4.69 | 7539336.66 |
| 42 | Caffeinol | C_20_H_28_O_3_ | [M+H]^+^ | 317.21 | 10.82 | 33616062.68 |
| 43 | Isorhamnetin | C_16_H_12_O_7_ | [M+H]^+^ | 317.07 | 6.46 | 1930112.37 |
| 44 | Ginkgoneolic acid | C_20_H_32_O_3_ | [M+H]^+^ | 321.24 | 11.45 | 8274443.51 |
| 45 | Isoform aconitin | C_20_H_27_NO3 | [M+H]^+^ | 330.21 | 6.89 | 17743758.07 |
| 46 | Columbin | C_20_H_22_O_6_ | [M+H]^+^ | 359.15 | 6.13 | 15304281.36 |
| 47 | Genistein B | C_19_H_18_O_7_ | [M+H]^+^ | 359.11 | 1.14 | 98701853.52 |
| 48 | Rosmarinic acid | C_18_H_16_O_8_ | [M+H]^+^ | 361.09 | 4.57 | 400615.12 |
| 49 | Curcumin | C_21_H_20_O_6_ | [M+H]^+^ | 369.13 | 9.96 | 5345129.23 |
| 50 | Aucubin | C_15_H_22_O_9_ | [M+H]^+^ | 369.12 | 2.21 | 3112265.84 |
| 51 | Forsytheins | C_21_H_24_O_6_ | [M+H]^+^ | 373.17 | 7.78 | 81425864.68 |
| 52 | Paraphyllin | C_22_H_33_NO4 | [M+H]^+^ | 376.25 | 4.94 | 21317926.78 |
| 53 | Loganic acid | C_16_H_24_O_10_0 | [M+H]^+^ | 377.15 | 4.69 | 61961252.11 |
| 54 | Eleutheroside | C_16_H_22_O_9_ | [M+H]^+^ | 381.12 | 2.97 | 39828726.65 |
| 55 | Pseudolaric acid C | C_21_H_26_O_7_ | [M+H]^+^ | 391.18 | 8.97 | 33835218.13 |
| 56 | Afzelin | C_21_H_20_O_10_0 | [M+H]^+^ | 433.11 | 6.37 | 1241906.35 |
| 57 | Astragalin | C_21_H_20_O_11_1 | [M+H]^+^ | 449.11 | 6.19 | 5731468.55 |
| 58 | Isosakuranin | C_22_H_24_O_10_0 | [M+H]^+^ | 449.15 | 3.63 | 58302445.74 |
| 59 | Asperuloside acid | C_18_H_24_O_12_2 | [M+H]^+^ | 455.12 | 4.25 | 1549292.02 |
| 60 | Berberine | C_26_H_30_O_7_ | [M+H]^+^ | 455.20 | 7.44 | 653736.80 |
| 61 | Forsythoside E | C_20_H_30_O_12_2 | [M+H]^+^ | 463.18 | 3.20 | 119659925.90 |
| 62 | Methylnissolin-3-O-glucoside | C_23_H_26_O_10_0 | [M+H]^+^ | 463.16 | 6.75 | 6053442.72 |
| 63 | Isoquercitrin | C_21_H_20_O_12_2 | [M+H]^+^ | 465.10 | 6.10 | 35289037.57 |
| 64 | Daphylloside | C_19_H_26_O_12_2 | [M+H]^+^ | 469.13 | 5.22 | 125152971.49 |
| 65 | Calceolarioside A | C_23_H_26_O_11_1 | [M+H]^+^ | 501.14 | 3.20 | 29042008.47 |
| 66 | Rutin | C_27_H_30_O_16_6 | [M+H]^+^ | 611.16 | 5.92 | 799317923.21 |
| 67 | Isorhamnetin-3-o-neohesperidin | C_28_H_32_O_16_6 | [M+H]^+^ | 625.18 | 6.45 | 3038686.75 |

Supplementary Material

| 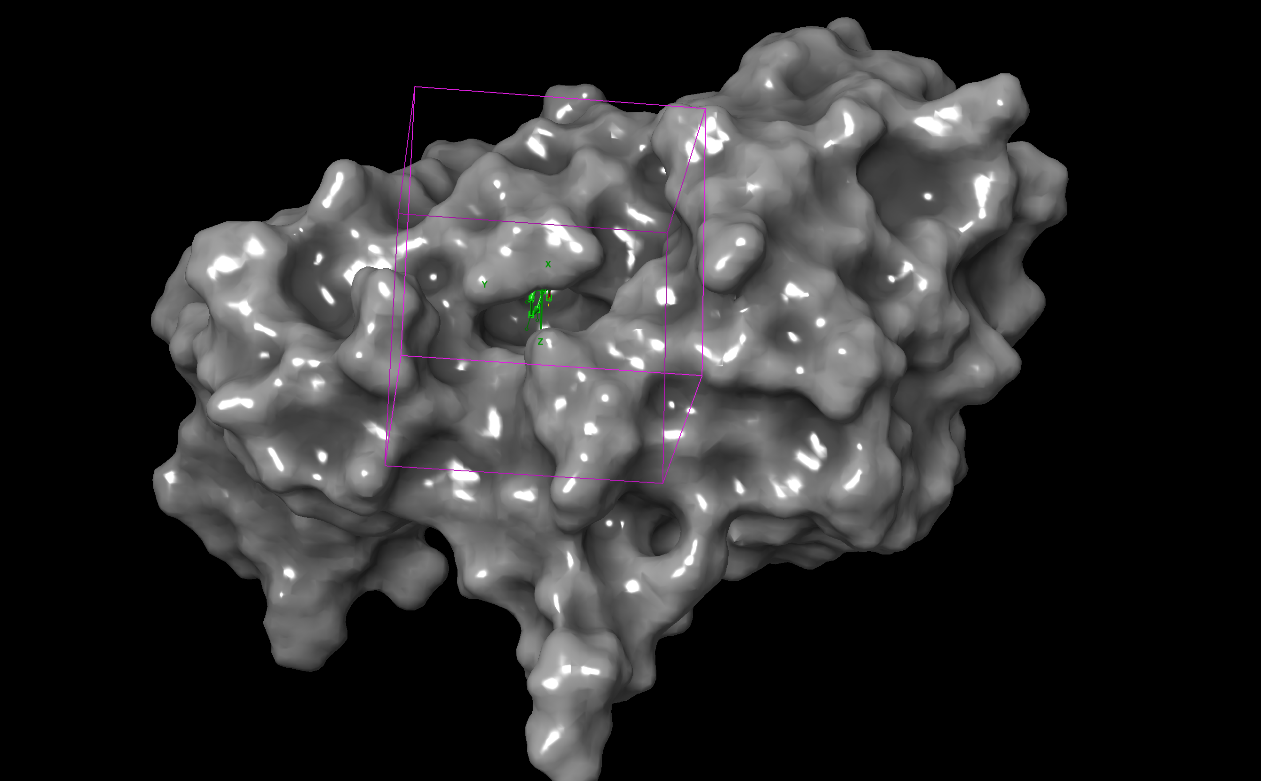 |
| --- |
| TP53 (PDB ID: 1YC5) |
| 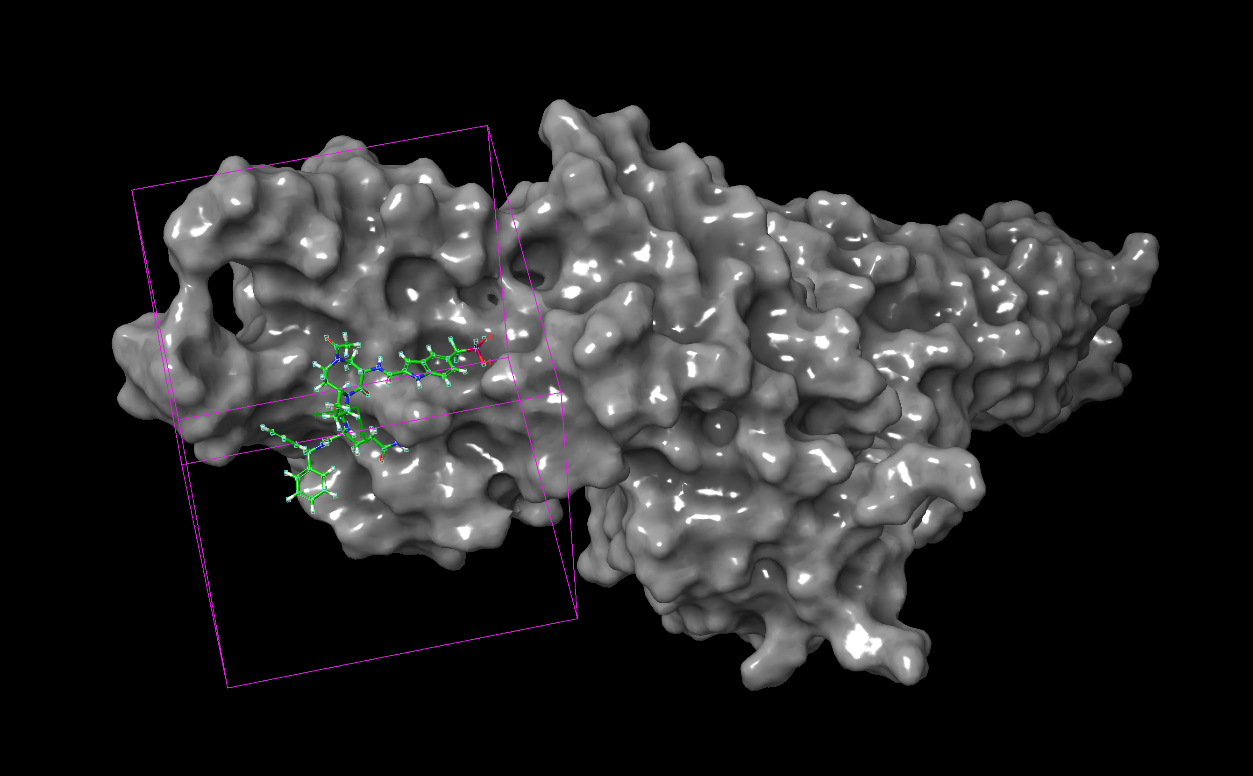 |
| STAT3（PDB ID: 6NJS） |
| 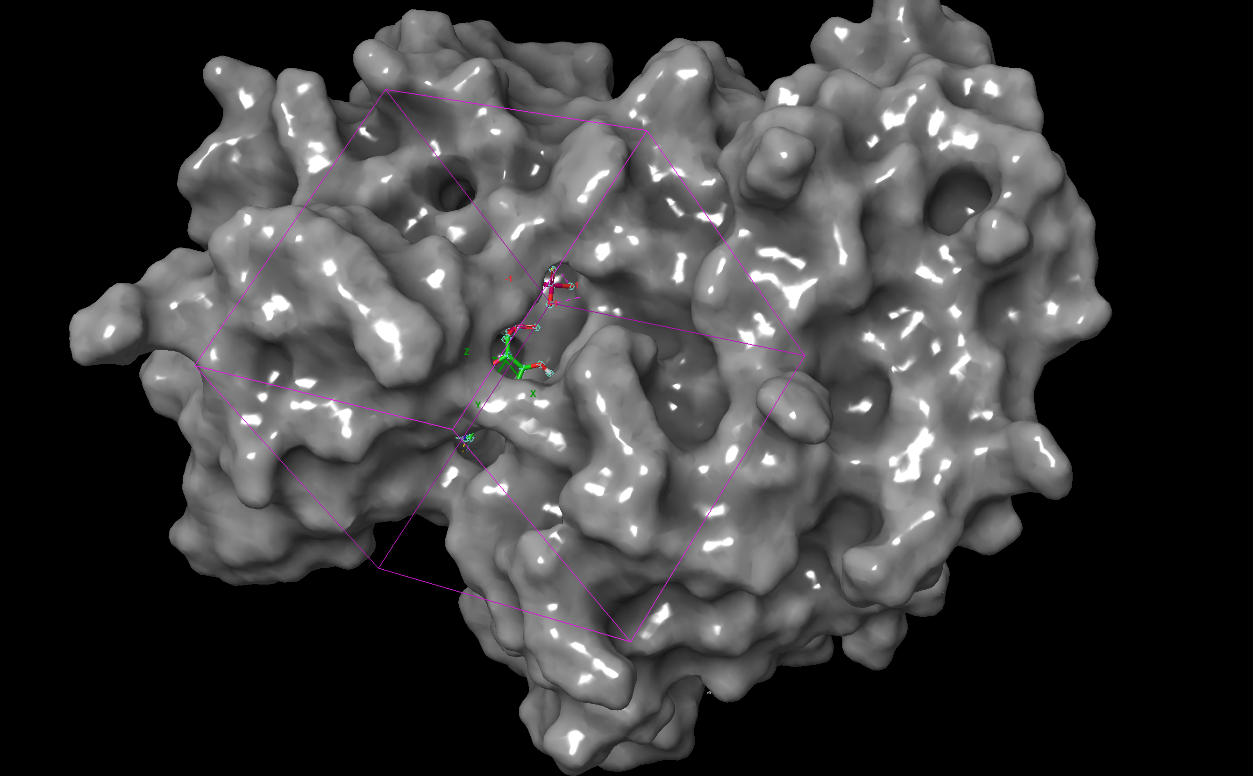 |
| MAPK1(PDB ID:2Y9Q) |
| 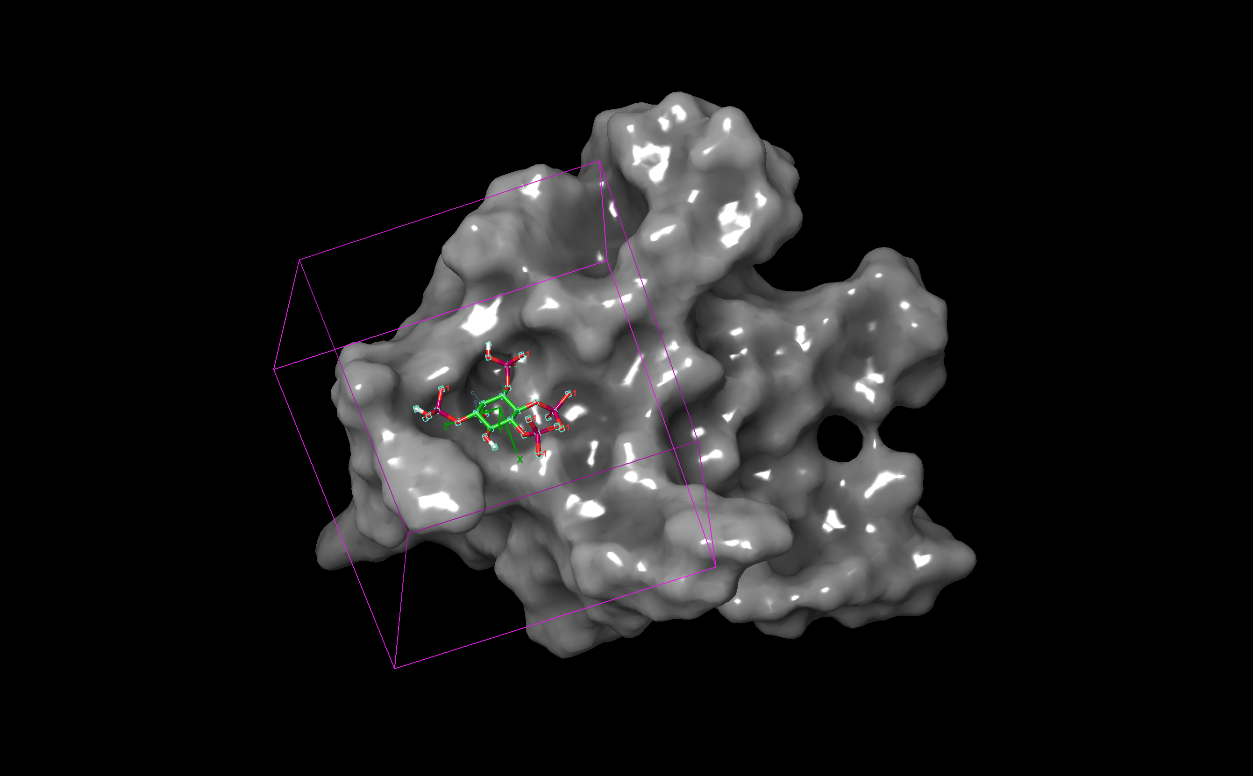 |
| AKT1(PDB ID: 1UNQ) |
| 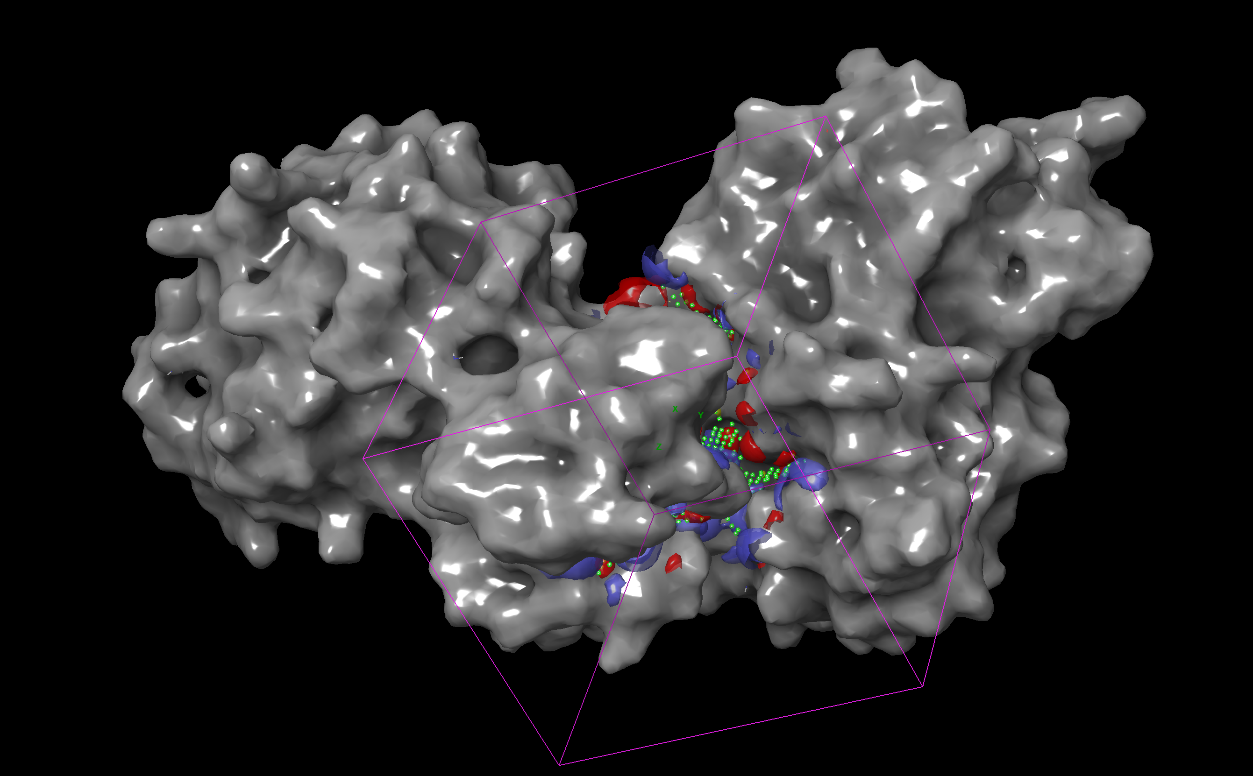 |
| MAPK3(PDB ID: 4QTB) |

**The optimal binding energy location of target protein.** The gray filling part is the target protein molecule, the ligand molecule or active region of the target protein is colored, and the red box is the best binding position of the target protein.
