# Supplementary material for: Prediction of the Active Components and Mechanism of Forsythia suspensa Leaf against Respiratory Syncytial Virus Based on Network Pharmacology
Source: Evid Based Complement Alternat Med. 2022 Jul 20;2022:5643345. doi: 10.1155/2022/5643345 (PMC9328944; doi:10.1155/2022/5643345)
Supplement: Supplementary Materials — Supplementary Table 1. Main chemical composition of FSL. The optimal binding energy location of the target protein. [file 5643345.f1.zip › 5643345.f1/Supporting documentations.pdf]

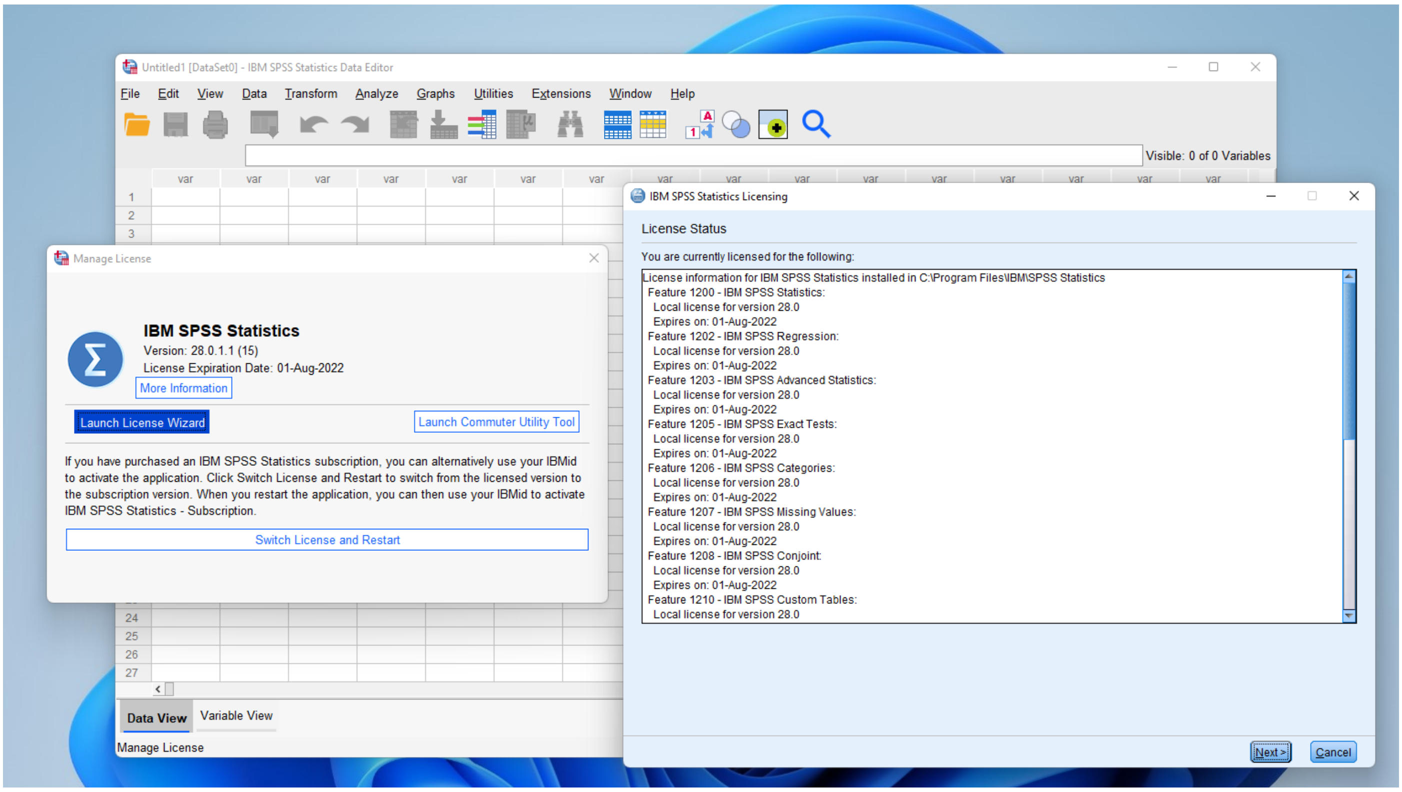

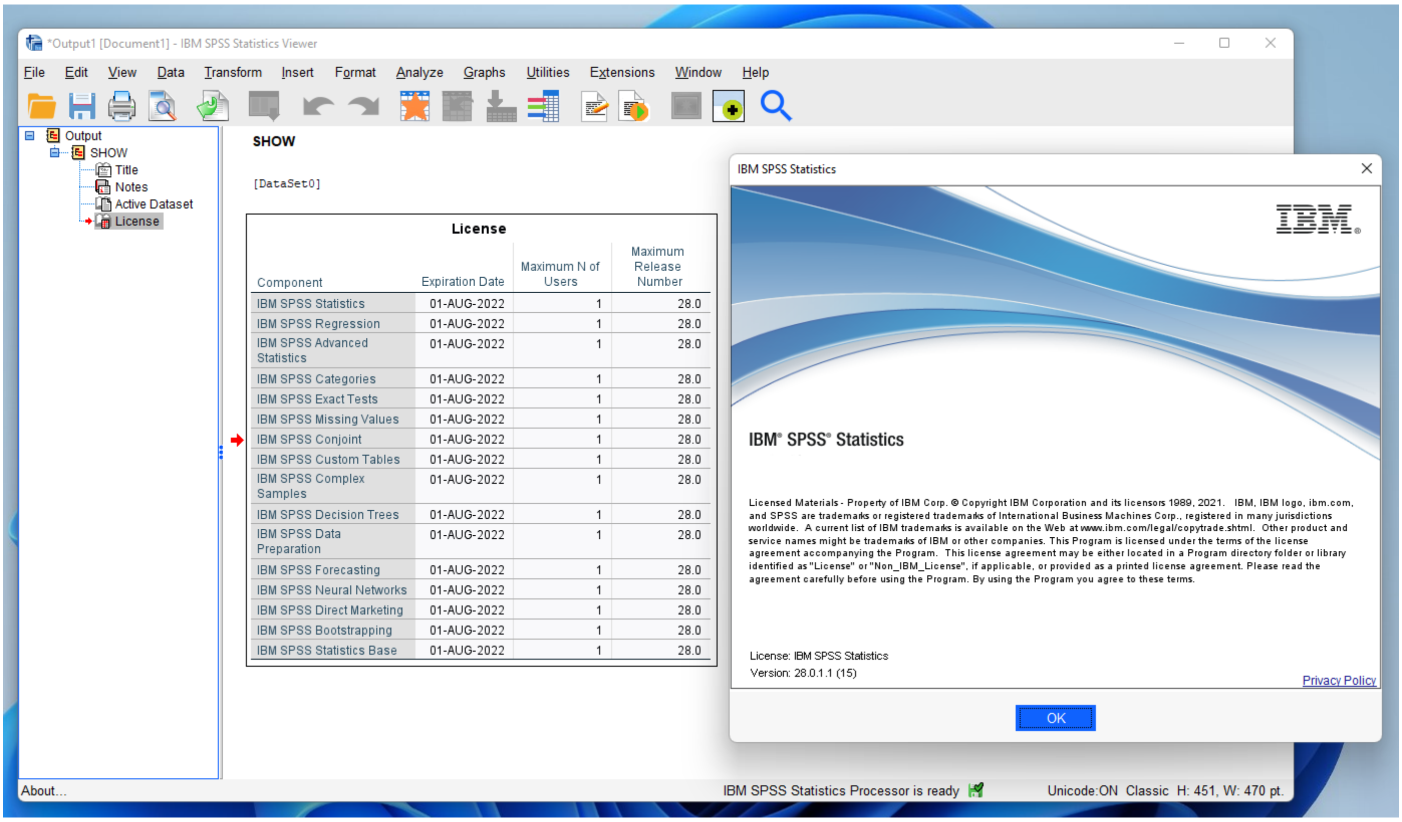

License

Component	Expiration Date	Maximum N of Users	Maximum Release Number
IBM SPSS Statistics	01-AUG-2022	1	28.0
IBM SPSS Regression	01-AUG-2022	1	28.0
IBM SPSS Advanced Statistics	01-AUG-2022	1	28.0
IBM SPSS Categories	01-AUG-2022	1	28.0
IBM SPSS Exact Tests	01-AUG-2022	1	28.0
IBM SPSS Missing Values	01-AUG-2022	1	28.0
IBM SPSS Conjoint	01-AUG-2022	1	28.0
IBM SPSS Custom Tables	01-AUG-2022	1	28.0
IBM SPSS Complex Samples	01-AUG-2022	1	28.0
IBM SPSS Decision Trees	01-AUG-2022	1	28.0
IBM SPSS Data Preparation	01-AUG-2022	1	28.0
IBM SPSS Forecasting	01-AUG-2022	1	28.0
IBM SPSS Neural Networks	01-AUG-2022	1	28.0
IBM SPSS Direct Marketing	01-AUG-2022	1	28.0
IBM SPSS Bootstrapping	01-AUG-2022	1	28.0
IBM SPSS Statistics Base	01-AUG-2022	1	28.0

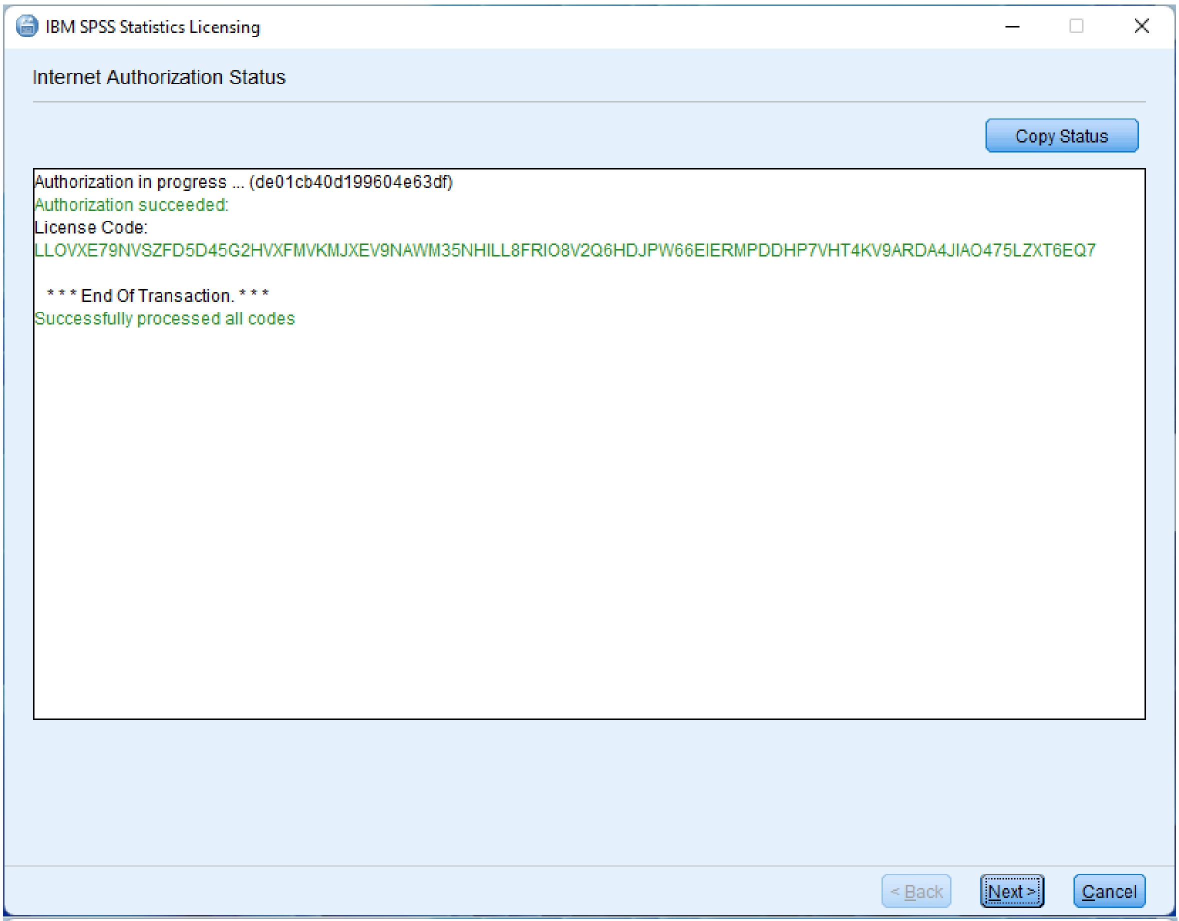
IBM SPSS Statistics Licensing
Internet Authorization Status
Copy Status
Authorization in progress ... (de01cb40d199604e63df)
Authorization succeeded:
License Code:
LLOVXE79NVSZFD5D45G2HVXFMVKMJXEV9NAWM35NHILL8FRIO8V2Q6HDJPW66EIERMPDDHP7VHT4KV9ARDA4JIAO475LZXT6EQ7
* * * End Of Transaction. * * *
Successfully processed all codes
< Back
Next >
Cancel

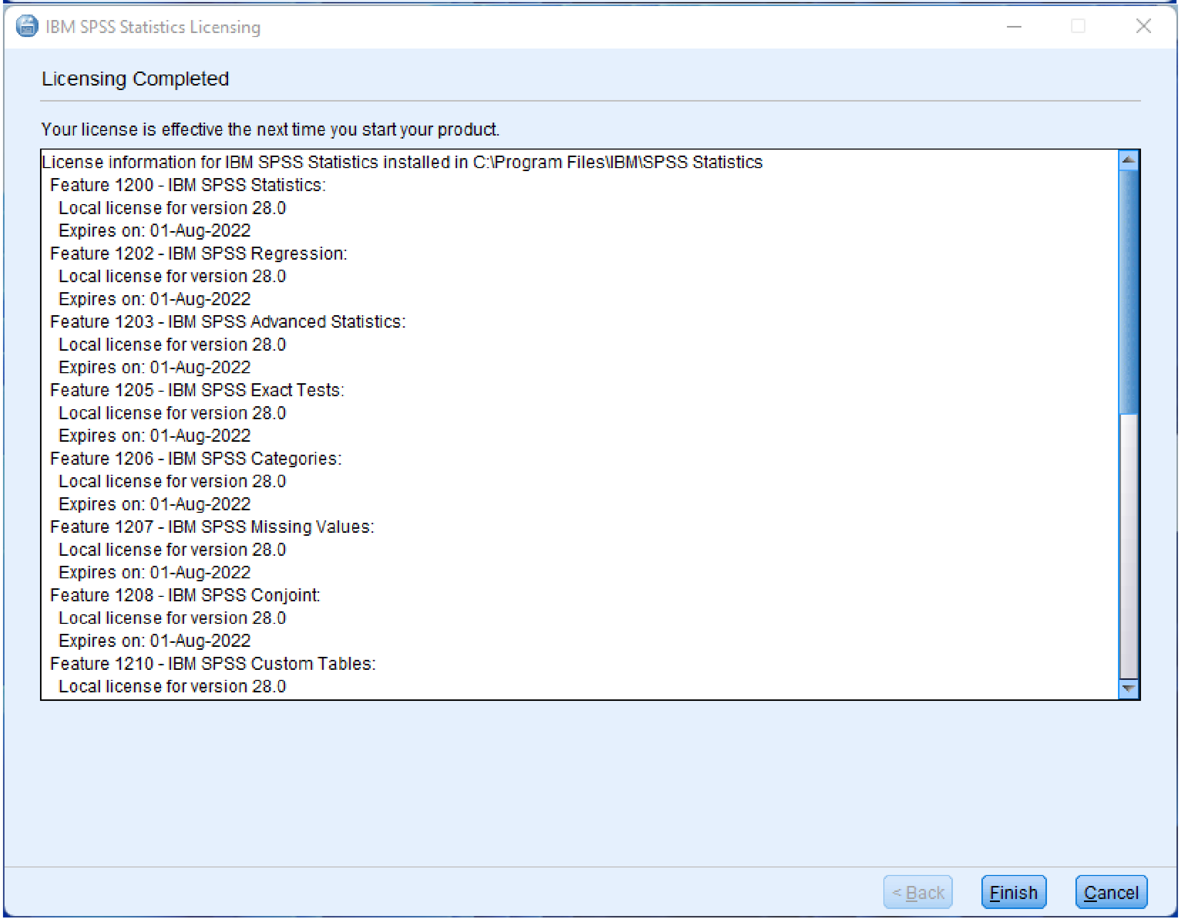
IBM SPSS Statistics Licensing
Licensing Completed
Your license is effective the next time you start your product.
License information for IBM SPSS Statistics installed in C:\Program Files\IBM\SPSS Statistics
Feature 1200 - IBM SPSS Statistics:
Local license for version 28.0
Expires on: 01-Aug-2022
Feature 1202 - IBM SPSS Regression:
Local license for version 28.0
Expires on: 01-Aug-2022
Feature 1203 - IBM SPSS Advanced Statistics:
Local license for version 28.0
Expires on: 01-Aug-2022
Feature 1205 - IBM SPSS Exact Tests:
Local license for version 28.0
Expires on: 01-Aug-2022
Feature 1206 - IBM SPSS Categories:
Local license for version 28.0
Expires on: 01-Aug-2022
Feature 1207 - IBM SPSS Missing Values:
Local license for version 28.0
Expires on: 01-Aug-2022
Feature 1208 - IBM SPSS Conjoint:
Local license for version 28.0
Expires on: 01-Aug-2022
Feature 1210 - IBM SPSS Custom Tables:
Local license for version 28.0
< Back
Finish
Cancel

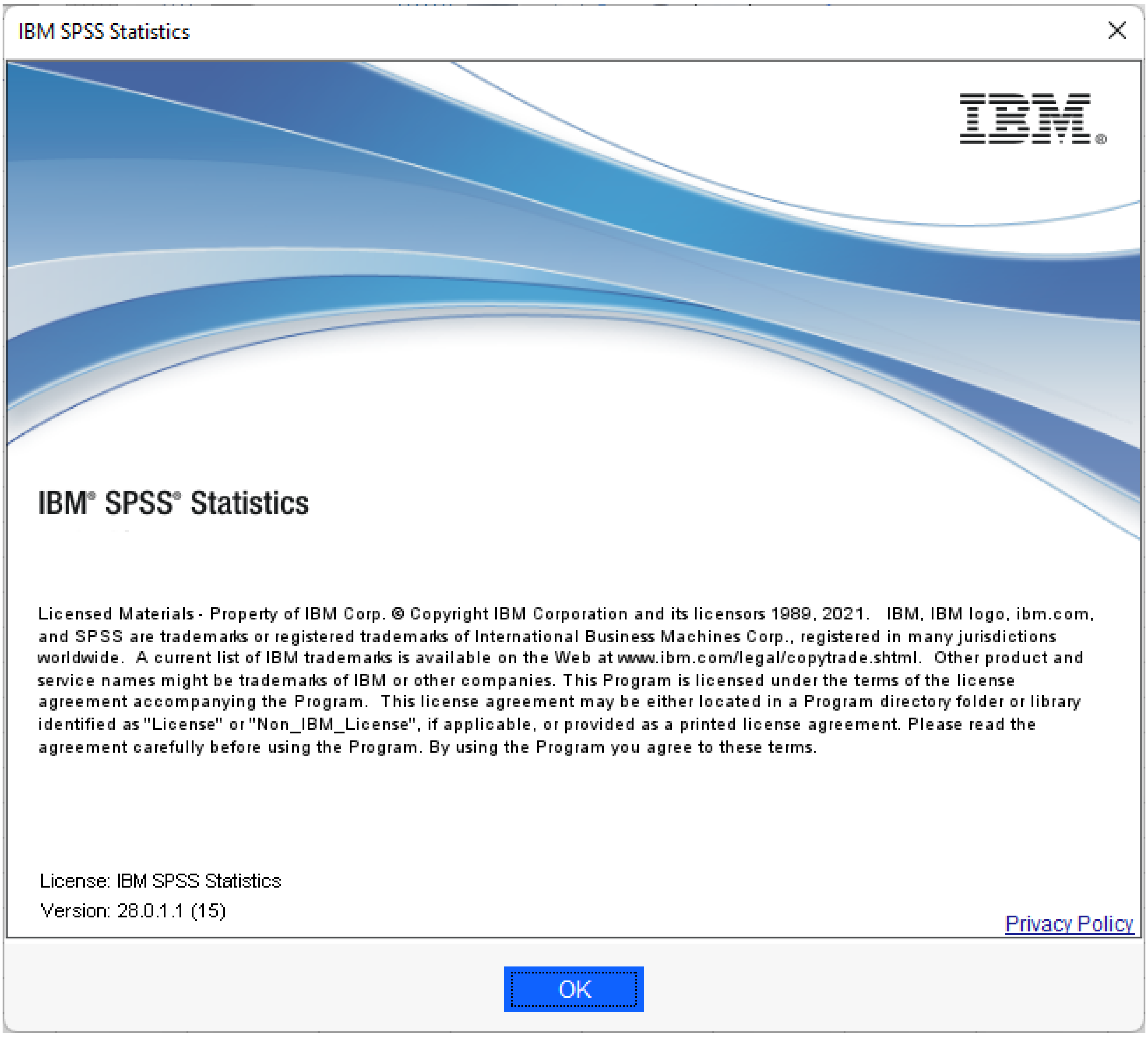
IBM SPSS Statistics
IBM
IBM® SPSS® Statistics
Licensed Materials - Property of IBM Corp. © Copyright IBM Corporation and its licensors 1989, 2021. IBM, IBM logo, ibm.com, and SPSS are trademarks or registered trademarks of International Business Machines Corp., registered in many jurisdictions worldwide. A current list of IBM trademarks is available on the Web at www.ibm.com/legal/copytrade.shtml. Other product and service names might be trademarks of IBM or other companies. This Program is licensed under the terms of the license agreement accompanying the Program. This license agreement may be either located in a Program directory folder or library identified as "License" or "Non_IBM_License", if applicable, or provided as a printed license agreement. Please read the agreement carefully before using the Program. By using the Program you agree to these terms.
License: IBM SPSS Statistics
Version: 28.0.1.1 (15)
Privacy Policy
OK

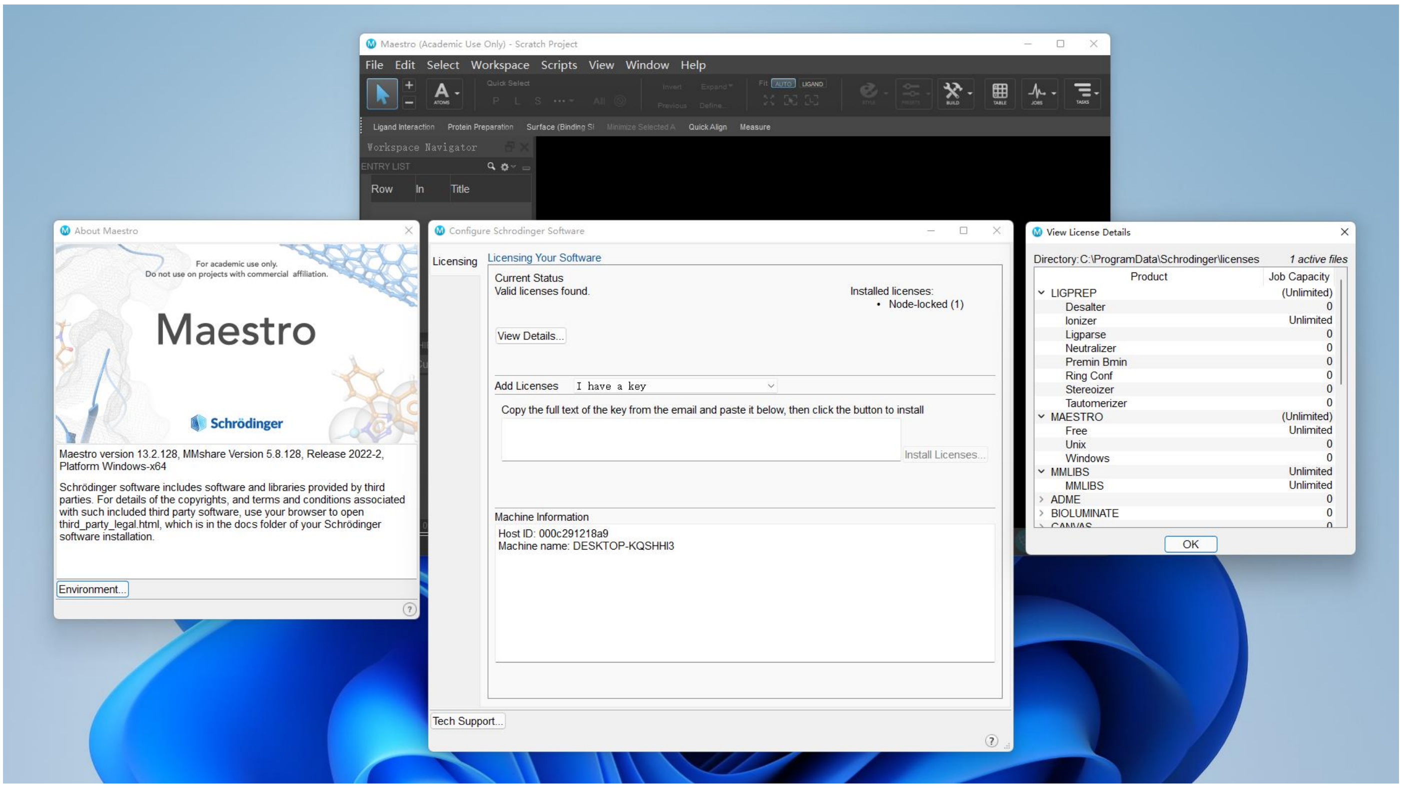
Maestro (Academic Use Only) - Scratch Project
File Edit Select Workspace Scripts View Window Help
Ligand Interaction Protein Preparation Surface (Binding Si Minimize Selected A Quick Align Measure
Workspace Navigator
ENTRY LIST
Row In Title
About Maestro
For academic use only.
Do not use on projects with commercial affiliation.
Maestro
Schrödinger
Maestro version 13.2.128, MMshare Version 5.8.128, Release 2022-2, Platform Windows-x64
Schrödinger software includes software and libraries provided by third parties. For details of the copyrights, and terms and conditions associated with such included third party software, use your browser to open third_party_legal.html, which is in the docs folder of your Schrödinger software installation.
Environment...
Configure Schrodinger Software
Licensing
Licensing Your Software
Current Status
Valid licenses found.
Installed licenses:
• Node-locked (1)
View Details...
Add Licenses I have a key
Copy the full text of the key from the email and paste it below, then click the button to install
Install Licenses...
Machine Information
Host ID: 000c291218a9
Machine name: DESKTOP-KQSHHI3
Tech Support...
View License Details
Directory: C:\ProgramData\Schrodinger\licenses
1 active files
Product
Job Capacity
LIGPREP (Unlimited)
Desalter 0
Ionizer Unlimited
Ligparse 0
Neutralizer 0
Premin Bmin 0
Ring Conf 0
Stereoizer 0
Tautomerizer 0
MAESTRO (Unlimited)
Free Unlimited
Unix 0
Windows 0
MMLIBS Unlimited
MMLIBS Unlimited
ADME 0
BIOLUMINATE 0
OK
